# The role of dynamic magnetic resonance imaging in exclusion of inguinal hernia in patients suffering from indefinitive groin pain

**DOI:** 10.1007/s10029-022-02642-6

**Published:** 2022-07-02

**Authors:** N. Rosbach, K. Wenger-Alakmeh, M. Lahrsow, S. Mahmoudi, S. Bernatz, T. J. Vogl, T. Schreckenbach, U. Pession, T. Gruber-Rouh

**Affiliations:** 1 Institute for Diagnostic and Interventional Radiology, University Hospital Frankfurt, Theodor-Stern-Kai 7, 60590 Frankfurt am Main, Germany; 2 Institute of Neuroradiology, University Hospital Frankfurt, Frankfurt am Main, Germany; 3 Departement of Surgery, University Hospital Frankfurt, Frankfurt am Main, Germany

**Keywords:** Dynamic MRI, MRI, Inguinal pain, Groin pain, Inguinal hernia

## Abstract

**Rationale and objectives:**

The objective of this study was to analyze the role of dynamic magnetic resonance imaging (MRI) in patients who suffered from groin pain and whose physical examination and ultrasound returned inconclusive/indefinite results, as well as in patients receiving an ongoing assessment for a previous herniotomy.

**Material and methods:**

For this study, 25 patients 14 women and 11 men were selected with a mean age of 41.6 years, including clinical complaints, such as groin pain and or a previous herniotomies. These patients underwent dynamic MRI. Reports were created by a radiology resident and a radiology consultant. Clinical and ultrasound documentation were compared to with imaging results from the MRI.

**Results:**

The results of the dynamic MRI were negative for 23 patients (92%) and positive for two patients (8%). One patient suffered from an indirect hernia and one from a femoral hernia. A repeated hernia was an excluding for the preoperated patients with pain and ongoing assessment.

**Conclusions:**

Dynamic MRI shows substantially higher diagnostic performance in exclusion of inguinal hernia, when compared to a physical examination and ultrasound. The examination can also be used in assessments to analyze the operation’s results.

## Introduction

With over 20 million groin hernia repairs worldwide per year, it is one of the most common operations performed in clinical routine [1]. Even though the rate of hernia accidents is relatively low, most patients with symptomatical inguinal hernia undergo hernia repair [2]. The typical clinical procedure consists of a physical examination and an ultrasound [3]. However, in some patients there is no definite diagnosis. According to the meta-analysis, an ultrasound has a high sensitivity of about 92% in diagnosing an inguinal hernia. On the other hand, it has a low specificity of 22.2% [4, 5]. Previous studies have shown that conventional dynamic magnetic resonance imaging (MRI) with regard to sensitivity and specificity is inferior to ultrasound [6]. To improve the patient’s outcome such as a reduction in chronic pain, a diagnosis must be accurate to determine the appropriate therapy. MRI examinations can therefore play an important role in diagnosis and influence the outcome for patients.

## Material and methods

This retrospective study was approved by the institutional review board (IRB). All patients gave informed consent for MRI.

Inclusion criteria included groin pain, indefinite findings in a physical examination, ultrasound, or assessment after an operation.

Exclusion criteria included underage patients and patients that were not able to give informed consent.

### Patient population

For this retrospective study, 25 patients with the mean age 41.6 years; 14 women (mean age: 38 years); and 11 men (mean age: 46 years]) were examined between May 2017 and October 2020. All patients underwent physical examination, and 7 patients (28%) underwent ultrasound prior to MRI examination. Seven patients (28%) were pre-operated with herniotomy due to inguinal hernia.

### MR imaging acquisition and examination

Examinations took place at the University Hospital Frankfurt am Main/Germany. We used three different MRI scanners, 1.5-T and 3-T MRI, in clinical routine using a standard 32-channel body coil (Magnetom Avanto^FIT^, Magnetom Aera, Magnetom Prisma^FIT^; Siemens Healthineers, Forchheim/Germany). MRI examinations were performed using four different sequences: (a) T2-weighted (T2w) half-Fourier acquisition single-shot turbo spin-echo (HASTE) in coronal, transversal, and sagittal orientation, (b) diffusion-weighted magnetic resonance imaging (DWI), (c) T1-weighted (T1w) volumetric interpolated breath-hold examination (VIBE) dixon in transversal orientation, and (d) T2w Fast imaging with steady precession (TRUFI) and provocation in coronal and sagittal orientation. Provocation was achieved by abdominal press of the patient.

### Image evaluation

Image evaluation was performed by using a conventional picture archiving and communication system station (PACS-station, Centricity Universal Viewer, Version 7.0). For establishing a standard of reverence, each examination was analyzed by a radiology resident and a senior attending (T.G.R., board-certified radiologist with 10 years of experience in abdominal imaging) with knowledge about the clinical examinations results, and a written finding was created.

After evaluation of the MRI series, four readers independently analyzed the static and dynamic MRI series. The readers were blinded to clinical examination results and written findings. Diagnostic confidence, image quality, and noise were evaluated by using a 5-point Likert scales (1, unacceptable; 5, excellent).

Four readers (K.W.A., radiology resident with 5 years of experience; M.L., radiology resident with 4 years of experience; S.B., radiology resident with 3 years of experience; S.M., radiology resident with 3 years of experience) analyzed separately the static and dynamic MRI series in a randomized blind study. First, static MRI series were assessed for the presence of inguinal herniation on a per-patient basis. After a 1-week interval, readers were asked to assess dynamic MRI series in the same way. Image ratings were conducted by using the above-mentioned 5-point Likert scales.

### Statistical analysis

Statistical analyses were performed using MedCalc 20.022 (MedCalc Software Ltd.). To assess the normality of data, the nonparametric Kolmogorov–Smirnov test was applied. Variables were expressed as means ± standard deviation and analyzed with the Wilcoxon test. A *p* < 0.05 was considered statistically significant. According to Landis and Koch, weighted *κ* statistics was used evaluating the interrater agreement [7].

## Results

During physical examination, no inguinal hernia could be found within the patients collective. Ten patients (40%) underwent an ultrasound examination. In six cases (24%), inguinal hernia was excluded. In four cases (16%), ultrasound showed an uncertain finding. In dynamic MRI examination, three inguinal hernias (12%) could be found in all patients with clinical and sonographical exclusion of inguinal hernia.

In one patient with an inconclusive ultrasound, a seroma was diagnosed with a dynamic MRI. All other patients were not diagnosed with inguinal hernia. The three patients with a pre-existing inguinal hernia in a dynamic MRI underwent herniotomy, with a good clinical outcome. Because of chronic inguinal pain, one patient underwent Lotheissen/McVay herniotomy, with exclusion of inguinal hernia afterwards. A 2-mm inguinal hernia was found in this patient. Therefore, this patient was declared as no herniation for the readers as the hernia without herniation is too small to detect with the MRI regarding the resolution. In dynamic MRI examinations with exclusion of inguinal hernia, two cases of femoral hernias, three cases of lymphadenopathy, five cases of musculoskeletal issues, one case of sigmadiverticulitis, and two cases of preperitoneal lipoma were diagnosed as origin of pain.

In dynamic MRI examinations, the sensitivity was 75% (3/4) and the specificity was 100% (22/22).

The other 22 patients with excluded inguinal hernia in dynamic MRI did not receive operations. Dynamic MRI led to different pain origins in cases where inguinal hernia was excluded, e.g., endometriosis extragenitalis, lymphadenopathy, sigmadiverticulitis, musculoskeletal issues. These patients were referred to different specialties regarding their diagnosis.

### Diagnostic accuracy

**Fig. 1:**
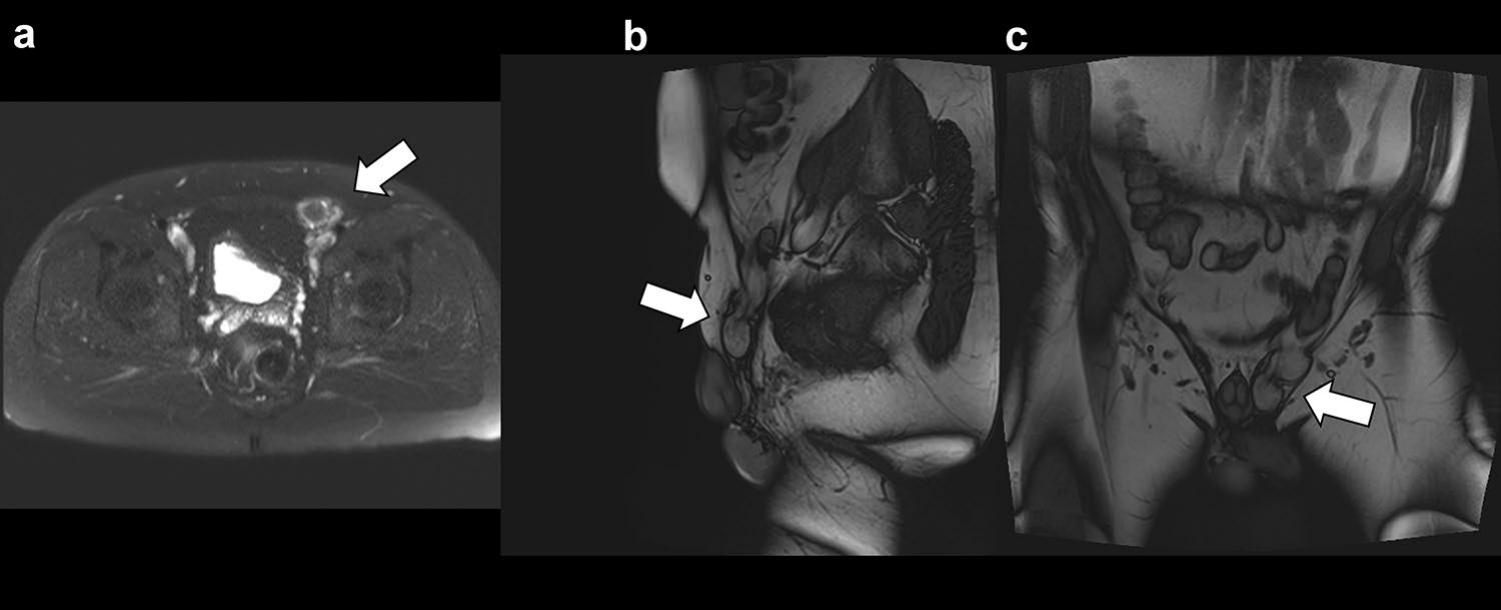
Case of a 32-year-old patient suffering from pain in the left groin for 1 day. Physical examination showed no certain sign of inguinal herniation. Ultrasound showed an uncertain formation in the left groin. For further investigation, a dynamic MRI examination was performed. Standard static MRI examination with axial T2-Haste sequence **a** and dynamic MRI examination with sagittal **b** and coronar **c** T2w. Fast imaging with steady precession (TRUFI) confirmed a herniation in the left groin

The MRI analysis showed a generally high sensitivity (12/12 [100%; 95% CI, 0.73–1.00] vs 12/12 [100%; 95% CI, 0.73–1.00]), specificity (85/88 [96%; 95% CI, 0.90–0.99] vs 86/88 [97%; 95% CI, 0.92–0.99]), and accuracy (97/100 [97%; 95% CI, 0.91–0.99] vs 98/100 [98%; 95% CI, 0.93–0.99]) of both static and dynamic MRI for the assessment of inguinal herniation. Interrater agreement was excellent for both static (*κ* = 0.96; 95% CI, 0.93–0.98) and for dynamic MRI (*κ* = 0.97; 95% CI, 0.95–0.99). Figure 1 shows an example case illustrating the potential for improving the detection of inguinal herniation in patients with uncertain clinical findings.

### Image ratings

**Fig. 2:**
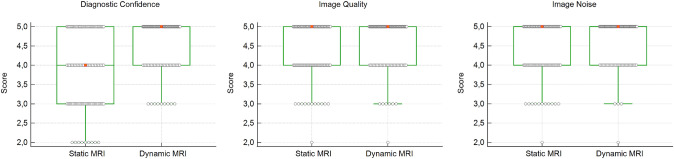
Box Whisker plot and dots illustrate rating results regarding the readers diagnostic confidence, image quality, and image noise of static and dynamic MRI. Median scores are shown as red boxes, and dots represent the score distribution. Diagnostic confidence was significantly higher in dynamic MRI compared to standard static MRI (*p* < 0.001). Ratings for image quality and image noise differed not significantly (*p* = 0.04)

Using MRI series, readers showed significant higher diagnostic confidence using dynamic MRI series (4.56 ± 0.66) vs using static MRI series (3.84 ± 1.0) (*p* < 0.0001) (Fig. 2). Interrater agreement was poor for static MRI (*κ* = 0.002; 95% CI, −0.39 to 0.39) and fair for dynamic MRI (*κ* = 0.39; 95% CI, 0.00–0.68).

Image noise was rated with mean scores of 4.4 ± 0.74 for static MRI and 4.6 ± 0.59 for dynamic MRI with no significant difference (*p* = 0.04).

Image quality varied not significantly (*p* = 0.04) between static (4.4 ± 0.72) and dynamic MRI (4.6 ± 0.65).

## Discussion

Dynamic MRI demonstrated high diagnostic accuracy in excluding inguinal hernias in comparison to a physical examination and ultrasound with patients with indefinite groin pain. In comparison to static MRI, there is a significant higher diagnostic confidence when using dynamic MRI. The high diagnostic accuracy in excluding inguinal hernias indicates the potential of dynamic MRI as a non-invasive, X-ray-free supplementing examination in patients with an inconclusive finding in ultrasound and physical examination where MRI is available. Physical examination and ultrasound are highly dependent on the examinators expertise, whereas dynamic MRI provides sufficient diagnosis in compliant patients.

Dynamic MRI resulted in a less frequent consultation of specialists by the patients and a more detailed treatment, as other pain origins than inguinal herniation could be detected.

Regarding the relatively low sensitivity, it must be mentioned that for one patient in laparotomy, the inguinal hernia was only 2 mm with no herniation of the bowel or fat. Therefore, the finding was too discrete for the MRI regarding the resolution. The undetected hernia could also not be detected retrospectively on a MRI. Thus, for clinical conspicuous inguinal hernia, dynamic MRI shows a higher sensitivity of up to 85% which has been confirmed in previous studies [8].

This study has several limitations, as dynamic MRI depends on the patient’s compliance. Dynamic MRI can be challenging in patients with incompliance due to insufficient abdominal pressure or patients moving, to exclude inguinal hernia. There are also clinical limitations as MRI is not available at every hospital and waiting periods for MRI examinations can be quite long.

## Conclusion

Inguinal hernia repair plays an important role in clinical routine. To avoid intervention related side effects, the diagnosis of an inguinal hernia must be accurate. In some patients, however, an ultrasound and a physical examination do not provide an accurate diagnosis. In these situations, dynamic MRI can play an important supportive role in the exclusion of inguinal hernias and therefore in minimizing the number of unnecessary operations.

## Abbreviations

MRI: Magnetic resonance imaging
IRB: Institutional review board
T2w: T2-weighted
HASTE: Half-Fourier acquisition single-shot turbo spin-echo
DWI: Diffusion-weighted magnetic resonance imaging
T1w: T1-weighted
VIBE: Volumetric interpolated breath-hold examination
TRUFI: T2w Fast imaging with steady precession
PACS station: Picture archiving and communication system station
